# Winter Wear

**Published:** 1874-11

**Authors:** 


### WINTER WEAR.

Although more persons die in summer than in winter, cold weather is particularly fatal to old people, and colds are taken by the people of all ages, which light up the quenchless fires of consumption, and all from the same cause—the want of sufficient and proper clothing.

All know that woolens are warmer than any other material. It is more correct to say that they "keep us warmer," for their value consists in keeping the heat about the body which comes from or is generated in the body, as they are bad conductors of heat, that is, carry it slowly away; so that woolen garments are not valuable in keeping the cold out, so much as keeping the warmth in.

The living body is of the same warmth the year round, whether at the tropics or at the poles, being about ninety-eight degrees, about one hundred and two under the armpits; a half-dozen degrees more or less make death pretty certain in a short time. Hence the preservation of health and life is intimately connected with a uniform temperature of the body, and as in health that temperature is measured by ninety-eight degrees, it should be the study of all to adjust the clothing to that end, always bearing in mind, however, that it is safer to be too warm than too cold. We can bear to be in a room for hours heated up to two hundred, but if kept in an apartment of forty degrees for a very short time, especially if damp, inflammation of the lungs and death in a week will be a very uniform result.

Real winter weather about New York city usually makes its advent a few days before Christmas; hence all old as well as all feeble persons should put on their thickest woolen undergarments from top to toe before the holidays. The best material is knitted; woven flannels "full up" by frequent washings, and become hard and impervious, confining the emanations unhealthily. These should be worn only in the day-time. If persons are very chilly, easily susceptible to cold or subject to night sweats, a thin flannel night gown should be worn next the skin; but persons in moderate or good health should wear muslin; and, in any event, no garment worn during the day should be used at night, but should have a plentiful airing.

There is one rule for all—wear sufficient clothing to keep off a feeling of chilliness when about your usual avocations; less than that subjects one to an attack of dangerous pneumonia any day or hour. More than that oppresses. Steadily aim by all possible ways and means to keep off a feeling of chilliness, for then a cold has been taken.

